# Editorial: Tuberculosis transmission: a battle between the host and the pathogen

**DOI:** 10.3389/fmicb.2026.1946986

**Published:** 2026-08-13

**Authors:** Krishna Kurthkoti, Vartika Sharma, Srinivasan Vijay

**Affiliations:** 1 School of Biosciences, Chanakya University, Bengaluru, India; 2 International Centre for Public Health, Rutgers, Newark, NJ, United States; 3 Department of Microbial Pathogenesis and Immunology, Texas A&M University, Naresh K. Vashisht College of Medicine, College Station, TX, United States

**Keywords:** Bedaquiline, drug resistance, epidemiology, *Mycobacterium tuberculosis*, tuberculosis

## Global TB surveillance, transmission dynamics, and novel therapeutics

Tuberculosis (TB), caused by *Mycobacterium tuberculosis* (*Mtb*), remains one of the leading causes of global mortality (World Health Organization, 2025). Transmission occurs primarily via aerosolized droplets (Acuña-Villaorduña et al., 2016). Current research into transmission dynamics is exploring diverse facets of the disease, including variations between low- and high-burden settings (Kamper-Jørgensen et al., 2012; Yates et al., 2016), the role of asymptomatic and subclinical cases (Dheda et al., 2025), transmission patterns between native and immigrant populations (Sandgren et al., 2014), and the impact of “superspreaders” (Melsew et al., 2019). Effective surveillance of these pathways is vital for identifying risk factors, detecting outbreaks, and monitoring the emergence of antibiotic-resistant strains.

Recent advancements in molecular technologies—particularly the transition from traditional genotyping methods to whole-genome sequencing (WGS)—have greatly enhanced our understanding of TB transmission (Nikolayevskyy et al., 2019). However, the adoption of these advanced surveillance tools is often constrained by economic factors and resource availability in developing regions (Mboowa, 2025).

## Insights from this Research Topic

Within this Research Topic, Liu et al. investigate the genotypic diversity and epidemiology of *Mtb* in Ningxia, China—a high-TB-burden region. Given local resource limitations, the authors employed spoligotyping (*n* = 115) and McSpoligotyping (*n* = 107) rather than WGS to analyze 222 isolates collected between 2005 and 2023. Their results demonstrate a clear dominance and increasing prevalence of Beijing family isolates across the two periods studied, rising from 78.26% (2005–2012) to 83.18% (2013–2023). Cluster analysis revealed three distinct groups using spoligotyping and two using McSpoligotyping, highlighting evolving localized transmission patterns.

In contrast, Bordoy et al. focus on Catalonia, Spain—a low-TB-burden region characterized by significant immigrant influxes. Utilizing WGS, they analyzed 783 isolates collected between December 2021 and June 2023. Their findings indicate that L4 sublineages (L4.1.2/Haarlem, L4.3/LAM, and L4.10/PGG3) dominate among Spanish-born individuals and long-term migrants (83%). Conversely, other lineages (L1/EAI, L2/Beijing, L3/CAS, and L4.1.1/X) are highly prevalent among recent migrants from India, Pakistan, China, and Peru.

These results underscore how migration and urbanization shape transmission structures. Notably, the authors also observed an association between specific lineages (L1/EAI, L3/CAS, and L4.10/PGG3) and extrapulmonary TB.

## Addressing the challenge of drug resistance

Tailored interventions may also alleviate the compounding challenges of drug resistance. The emergence and spread of resistance to frontline antibiotics pose a severe threat to global health, necessitating the urgent development of novel therapeutics (Motta et al., 2024). Standard drug-susceptible TB therapy involves a strict 6-month regimen combining four major antibiotics (Motta et al., 2024):

- Isoniazid
- Rifampicin
- Ethambutol
- Pyrazinamide.

Bedaquiline (BDQ), a promising anti-TB agent that targets mycobacterial ATP synthesis, has been utilized since 2012, particularly for drug-resistant cases. However, emerging resistance to BDQ—mapped primarily to mutations in the *rv0678, atpE*, and *pepQ* genes—threatens its long-term clinical efficacy (Sonnenkalb et al., 2023).

Addressing this issue, Malope et al. reported a cross-sectional study from Limpopo, South Africa. Among 147 drug-resistant *Mtb* isolates tested for BDQ susceptibility, 128 remained sensitive, while 19 exhibited resistance (minimum inhibitory concentration > 1 μg/mL. WGS analysis revealed that the vast majority of resistant strains (14/19; 89.1%) harbored mutations in *rv0678*. These isolates spanned four lineages: East African–Indian, East Asian (including Beijing), Indo-Oceanic, and Euro-American. Notably, the Euro-American lineage accounted for the largest proportion of BDQ-resistant strains (62.5%). Despite the localized sample size, this study underscores the critical importance of concurrent phenotypic and molecular surveillance to ensure the early detection and management of drug resistance.

## Role of basic research in *Mtb* biology

### Pathogenesis and vertical transmission

Fonseca-Perez et al. examine the risk of vertical transmission and the progression from latent to active TB during pregnancy. Using the standard laboratory strain H37Rv in both logarithmic and reactivated dormant phases (NRP1 and NRP2), they performed *ex vivo* infections of human placental explants.

Their findings demonstrate that *Mtb* reactivated from dormancy readily infects and replicates within placental tissue, causing significant histopathological changes including:

- Villitis
- Stromal fibrosis
- Reduced vascular integrity (particularly severe with NRP2-stage bacteria).

The study also noted a significant upregulation of reactivation (*rpfB*), dormancy (*dosR, hspX, icl1*), and stress-response genes (*sigH, whiB3*), alongside heightened metalloproteinase activity. These results highlight the risks of latency reactivation during pregnancy, emphasizing the clear potential for severe placental damage and mother-to-child transmission.

## Conclusion

Overall, the contributions featured in this Research Topic underscore the absolute necessity for robust molecular surveillance to track transmission dynamics, identify active clusters, and monitor the emergence and spread of drug resistance. The integration of rapid molecular diagnostics and whole-genome sequencing is proving to be a fundamental toolkit for decoding the epidemiology of TB across diverse geographic and demographic landscapes. Continued investment in surveillance infrastructure and translational research remains imperative to advance global TB control, inform progressive public health policies, and ultimately mitigate the burden of this enduring disease.
